# Trends in Use of Single- vs Dual-Chamber Implantable Cardioverter-Defibrillators Among Patients Without a Pacing Indication, 2010-2018

**DOI:** 10.1001/jamanetworkopen.2022.3429

**Published:** 2022-03-22

**Authors:** Ryan T. Borne, Paul Varosy, Zhou Lan, Frederick A. Masoudi, Jeptha P. Curtis, Daniel D. Matlock, Pamela N. Peterson

**Affiliations:** 1 Division of Cardiology, University of Colorado Health, Colorado Springs; 2 Division of Cardiology, University of Colorado Anschutz Medical Campus, Aurora; 3 Cardiology Section, Rocky Mountain Regional Veterans Affairs Medical Center, Aurora, Colorado; 4 Center for Outcomes Research and Evaluation, Yale School of Medicine, New Haven, Connecticut; 5 Research and Analytics, Ascension Health, St Louis, Missouri; 6 Division of Cardiology, Yale University School of Medicine, New Haven, Connecticut; 7 Department of Medicine, University of Colorado Anschutz Medical Campus, Aurora; 8 Veterans Affairs Eastern Colorado Geriatric Research Education and Clinical Center, Denver; 9 Division of Cardiology, Denver Health Hospital, Denver, Colorado

## Abstract

**Importance:**

Use of dual-chamber implantable cardioverter-defibrillator (ICD) systems among patients without a pacing indication is an example of low-value care given higher procedural risks, higher costs, and little evidence for benefit from an atrial lead. However, variation in the use of dual-chamber systems was present among patients without a pacing indication.

**Objective:**

To examine the temporal trends and hospital variation in use of single- and dual-chamber ICD implantation among patients without a pacing indication undergoing first-time ICD implantation.

**Design, Setting, and Participants:**

A multicenter cross-sectional study was conducted using the US National Cardiovascular Data Registry ICD Registry. A total of 266 182 patients undergoing initial implantation of a single- or dual-chamber transvenous ICD without a bradycardia pacing indication, class I or II cardiac resynchronization therapy indication, or history of atrial fibrillation or atrial flutter were included. The study was conducted from April 1, 2010, to December 31, 2018; data analysis was performed from October 19, 2020, to January 5, 2022.

**Exposures:**

Implantation of a single- or dual-chamber ICD.

**Main Outcomes and Measures:**

Temporal trends among patients undergoing single- vs dual-chamber ICDs were determined using the Cochran-Armitage trend test, and hospital-level variation using adjusted hospital median odds ratios was examined.

**Results:**

A total of 266 182 patients (single-chamber ICD, 134 925; dual-chamber ICD, 131 257) were included in this analysis; mean (SD) age was 58.0 (14.0) years and 91 990 patients (68.2%) were men. The use of dual-chamber ICDs decreased from 64.7% (n = 15 694) in 2010 to 42.2% (n = 9762) in 2018 (*P* < .001). Adjusted for patient characteristics, the median hospital-level proportion of single-chamber ICDs increased from 42.9% (95% CI, 42.6%-45.0%) in 2010 to 50.0% (95% CI, 47.8%-51.0%) in 2018. The median odds ratio for the use of dual-chamber ICDs, adjusted for patient characteristics, was 1.6 (95% CI, 1.6-1.8) in 2010 and 1.5 (95% CI, 1.5-1.8) in 2018, indicating decreasing but persistent variation in use.

**Conclusions and Relevance:**

In this national study of US patients undergoing first-time ICD implantation without a clinical indication for an atrial lead, the use of dual-chamber devices decreased. However, institutional variability in the use of atrial leads persists, suggesting differences in individual or institutional cultures of real-world practice and opportunity to reduce this low-value practice.

## Key Points

**Question:**

What are the trends and variability in use of single- and dual-chamber implantable cardioverter-defibrillators (ICDs) among patients undergoing ICD implantation without a pacing indication?

**Findings:**

In this cross-sectional study of 266 182 patients undergoing first-time ICD implantation without a pacing indication, the use of dual-chamber ICDs decreased. However, there was persistent variation in use of the devices despite adjustment for patient characteristics.

**Meaning:**

In this cross-sectional study of patients in contemporary practice undergoing first-time ICD implantation without a pacing indication, institutional variability in the use of atrial leads appears to persist, suggesting differences in individual or institutional cultures of real-world practice and opportunity to reduce this low-value practice.

## Teaser

This cross-sectional study compares the use of single- vs dual-chamber implantable cardioverter-defibrillators over time in patients without an indication for an atrial lead.

## Introduction

Although most patients enrolled in clinical trials assessing the mortality benefit of implantable cardioverter-defibrillators (ICDs) received a single-chamber device, wide variation exists in the use of dual-chamber systems in these patients in contemporary real-world practice.^1,2,3,4^ Current guidelines do not specify single- or dual-chamber devices in the absence of a pacing indication; however, guidelines and expert consensus do not recommend an atrial lead in the absence of atrial arrhythmias or need of pacing.^5,6^

The addition of an atrial lead can offer theoretical benefits, but there has been little evidence suggesting that it provides meaningful improvements in patient-centered outcomes for those who do not need atrial pacing. A 2017 longitudinal study found no association between the use of an atrial lead and lower risk of inappropriate shock.^7^ In contrast, the addition of an atrial lead was found to be associated with an increase in risk of periprocedural complications and, in some reports, an increased risk in mortality.^8,9^ Thus, use of dual-chamber devices in patients without a pacing indication is an example of low-value health care. Conversely, use of single-chamber devices is less expensive, associated with fewer complications, and thus an example of high-value care. Yet, earlier studies reported substantial variation in the use of single- and dual-chamber systems, which is not explained by patient characteristics but by the implanting center.^4,9^

The extent to which more recent guidelines and appropriate use criteria have been incorporated into contemporary practice are unclear. The National Cardiovascular Data Registry (NCDR) ICD Registry provides a unique resource for evaluating the current practice of ICD implantation in a contemporary population. Accordingly, we sought to examine the temporal trends in use and variation of single- and dual-chamber ICD implantation among patients undergoing first-time ICD implantation without a pacing indication.

## Methods

### Data Source

For this cross-sectional study, data were obtained from the NCDR ICD Registry; the details have been reported previously.^10^ The ICD Registry was designed to satisfy the requirements of the 2005 Centers for Medicare & Medicaid Services coverage with evidence development for primary prevention ICD implantation. In addition to expanded coverage, Centers for Medicare & Medicaid Services mandated that data on all Medicare primary prevention implants be entered into the NCDR ICD Registry until the data collection requirement ended on February 15, 2018.

The registry collects data from more than 1500 hospitals in the US and included more than 1.3 million records as of the end of 2014.^11,12,13^ Although the Centers for Medicare & Medicaid Services mandated that only primary prevention devices be entered into the registry, it is estimated that 90% of all ICD implantations are documented.^11^ The registry uses both a standardized data set and definitions, has requirements in place to ensure uniform data entry and transmission, and is subject to data quality checks.^14^ All data submissions are evaluated for errors and completeness. This information is summarized in an automated report that is sent to the participants after each submission of information. The NCDR audit program, which includes hospital medical record reviews and blinded data abstractions, serves as an additional mechanism to assess the accuracy of the data and enables participants to identify areas for improved data entry. Statistical analysis in this study was approved and completed by the Yale Center for Outcomes Research and Evaluation. The initial analysis was performed on October 19, 2020. Data analysis was conducted from October 19, 2020, to January 5, 2022. Analyses of the NCDR ICD Registry were performed under an institutional review board approval by Yale University, with a waiver of informed consent because of the study design. This study followed the Strengthening the Reporting of Observational Studies in Epidemiology (STROBE) reporting guideline for cross-sectional studies.

### Study Population

All patients undergoing initial implantation of a single- or dual-chamber ICD in the NCDR ICD Registry were included from April 2010 to December 2018 (versions 2.1 and 2.2). Demographic characteristics were obtained as given on the NCDR ICD Registry input form, including race and ethnicity (Black or African American, Hispanic, White, and other [grouped as Asian, American Indian or Alaskan Native, and Native Hawaiian or Pacific Islander]). Patients were excluded if they had received a prior ICD, pacemaker, coronary sinus/left ventricular or epicardial lead, if the index procedure was a generator change or lead revision procedure, subcutaneous ICD, a class I or II guideline bradycardia pacemaker indication (version 2.1: bradycardic arrest or second- or third-degree heart block; version 2.2: indication for permanent pacing), class I indication (left ventricular ejection fraction ≤35%, left bundle branch block with QRS>150 ms with New York Heart Association [NYHA] class II-IV heart failure), class 2 indication (left ventricular ejection fraction <35%, left bundle branch block with QRS 120-149 ms, NYHA class II-IV heart failure or non–left bundle branch block with QRS 120-149 ms, and NYHA class III or IV heart failure or non–left bundle branch block with QRS≥150 ms, and NYHA class II-IV heart failure, or left ventricular ejection fraction ≤30% and left bundle branch block with QRS>150 ms, coronary disease, and NYHA class I heart failure), cardiac resynchronization therapy indication, or a history of atrial fibrillation or atrial flutter.^5^ Hospitals were restricted to a minimum threshold of 10 procedures per year (scaled from 2010 data).

### Statistical Analysis

Baseline characteristics at the time of implantation were compared between patients who underwent single- or dual-chamber ICD implantation, using χ^2^ tests for categorical variables and *t* tests for continuous variables. Continuous variables are presented as means (SDs) and categorical variables as frequency and percent.

To determine temporal changes in single- vs dual-chamber systems, we examined the frequency of implantation over the years. For each year, we calculated the proportions of patients receiving single- vs dual-chamber systems and plotted them over the years. The Cochran-Armitage trend test was used to identify a temporal trend.

The hospital-level variation is expressed by the hospital-level distribution of the percentages of single-chamber ICD use and the median odds ratio (mOR), which describes the likelihood that the treatment of a patient with similar comorbidities would differ at 2 randomly selected hospitals.^15^ The hospital-level distribution is a method to show the variation, and the mOR further summarizes the variation in the widely used OR scale. The mOR is always greater than or equal to 1.0, with an mOR of 1.0 suggesting no variation between hospitals and higher mORs indicate greater variation. Both the hospital-level distribution and the mOR were adjusted for patient characteristics using a logistic regression model. The covariates in the logistic regression model include all the patient characteristics listed in Table 1, excluding hypertension and prior heart failure hospitalization because these data were no longer collected in version 2.2 of the ICD Registry. All the covariates included in the model had missing values of less than 0.2%. We used SAS, version 9.4 TS Level 1M5 (SAS Institute Inc) for statistical analysis. No extension packages were used. Findings were considered significant at *P* < .05. The hypothesis tests were 2-sided.

**Table 1.** Baseline Characteristics Among Patients Undergoing Single- vs Dual-Chamber ICD Implantation

Characteristic	No. (%)		*P* value
	Single-chamber ICD (n = 134 925)	Dual-chamber ICD (n = 131 257)	
**Patient characteristics**			
Age, mean (SD), y			
All	58.0 (14.0)	62.5 (13.2)	<.001
65-74	69.0 (2.8)	69.2 (2.8)	<.001
75-84	78.4 (2.7)	78.7 (2.8)	<.001
>84	87.3 (3.9)	87.1 (3.5)	.05
Sex			
Male	91 990 (68.2)	94 316 (71.9)	<.001
Female	42 935 (31.8)	36 941 (28.1)	
Race and ethnicity			
Black or African American	32 088 (23.8)	22 082 (16.8)	<.001
Hispanic	6198 (4.6)	6767 (5.2)	
White	80 506 (59.7)	90 321 (68.8)	
Other^a^	16 133 (12.0)	12 087 (9.2)	
Ischemic cardiomyopathy	n = 134 692	n = 131 001	
Yes	83 606 (62.1)	92 400 (70.5)	<.001
No	51 086 (37.9)	38 601 (29.5)	
History of heart failure	n = 134 869	n = 131 179	
Yes	103 306 (76.6)	89 961 (68.6)	<.001
No	31 563 (23.4)	41 218 (31.4)	
History of heart failure hospitalization	n = 67 667	n = 67 419	
Yes	32 525 (48.1)	29 960 (44.4)	<.001
No	35 142 (51.9)	37 459 (55.6)	
NYHA functional classification	n = 102 888	n = 89 596	
I	7089 (6.9)	6445 (7.2)	<.001
II	54 032 (52.5)	43 571 (48.6)	
III	39 738 (38.6)	37 766 (42.2)	
IV	2029 (2.0)	1814 (2.0)	
Cerebrovascular disease	n = 134 818	n = 131 118	
Yes	15 626 (11.6)	17 468 (13.3)	<.001
No	119 192 (88.4)	113 650 (86.7)	
Diabetes	n = 134 824	n = 131 139	
Yes	50 566 (37.5)	47 164 (36.0)	<.001
No	84 258 (62.5)	83 975 (64.0)	
Current dialysis	n = 134 820	n = 131 125	
Yes	3918 (2.9)	3156 (2.4)	<.001
No	130 902 (97.1)	127 969 (97.6)	
Chronic lung disease	n = 134 824	n = 131 117	
Yes	24 640 (18.3)	24 848 (19.0)	<.001
No	110 184 (81.7)	106 269 (81.0)	
Hypertension	n = 92 004	n = 101 182	
Yes	69 308 (75.3)	79 581 (78.7)	<.001
No	22 696 (24.7)	21 601 (21.3)	
QRS interval, ms	n = 134 925	n = 131 257	
≤120	128 022 (94.9)	118 606 (90.4)	<.001
>120	6903 (5.1)	12 651 (9.6)	
Left ventricular ejection fraction (SD), %	30 (13.1)	33.1 (14.3)	<.001
Hemoglobin, mean (SD), g/dL	13.1 (2.1)	13.1 (2.0)	<.001
Creatinine, mean (SD), mg/dL	1.3 (1.2)	1.2 (1.1)	<.001
Sodium, mean (SD), mEq/L	138.7 (4.4)	138.8 (4.6)	<.001
**Hospital characteristics**			
Location			
Rural	14 751 (10.9)	14 497 (11.0)	<.001
Suburban	37 718 (28.0)	38 362 (29.2)	
Urban	82 456 (61.1)	78 398 (59.7)	
Type	n = 134 925	n = 131 257	
Government	2726 (2.0)	2077 (1.6)	<.001
Private/community	107 717 (79.8)	112 952 (86.1)	
University	24 482 (18.1)	16 228 (12.4)	
Region			
Midwest	29 713 (22.1)	32 064 (24.5)	<.001
Northeast	29 157 (21.7)	18 874 (14.4)	
South	59 582 (44.3)	59 456 (45.3)	
West	16 053 (11.9)	20 736 (15.8)	
Adult EP training	n = 91 331	n = 100 523	
Yes	65 546 (71.8)	68 724 (68.4)	<.001
No	25 785 (28.2)	31 799 (31.6)	
HRS training	n = 91 331	n = 100 523	
Yes	28 008 (30.7)	32 206 (32.0)	<.001
No	63 323 (69.3)	68 317 (68.0)	
Thoracic surgery training	n = 91 331	n = 100 523	
Yes	1259 (1.4)	1773 (1.8)	<.001
No	90 072 (98.6)	98 750 (98.2)	

Abbreviations: EP, electrophysiologist; HRS, Heart Rhythm Society; ICD, implantable cardioverter-defibrillator; NYHA, New York Heart Association.

SI conversion factor: To convert creatinine to micromoles per liter, multiply by 88.4; hemoglobin to grams per liter, multiply by 10; sodium to millimoles per liter, multiply by 10.

^a^ Included Asian, American Indian/Alaskan Native, and Native Hawaiian/Pacific Islander.

## Results

### Study Population

**Figure 1.**
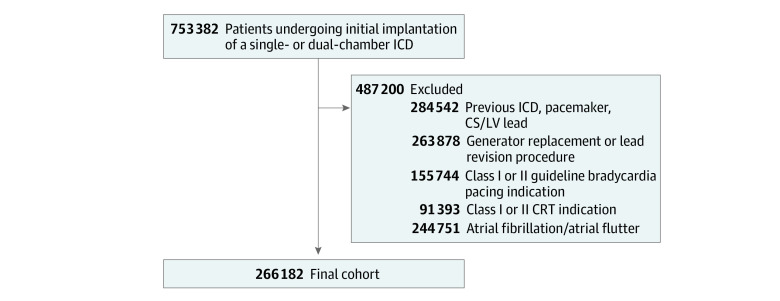
Criteria for Analysis of Patients Receiving Implantable Cardioverter-Defibrillators (ICDs) CRT indicates cardiac resynchronization therapy; CS, coronary sinus; LV, left ventricular.

Within the NCDR ICD Registry, data are available on 753 382 patients who underwent a primary or secondary prevention ICD implantation between April 1, 2010, and December 31, 2018. For this study, we excluded patients with the following status: prior ICD, pacemaker, or coronary sinus/left ventricular lead (n = 284 542); generator replacement or lead revision procedure (n = 263 878); class I or II guideline bradycardia pacing indication (n = 155 744); class I or II indication for cardiac resynchronization therapy (n = 91 393); and a history of atrial fibrillation or atrial flutter (n = 244 751), resulting in a study cohort of 266 182 patients (Figure 1).

### Patient, Hospital, and Implanter Demographic Characteristics

Characteristics of the patient population are presented in Table 1. A total of 131 257 patients (49.3%) underwent dual-chamber and 134 925 patients (50.7%) underwent single-chamber ICD implantation. Patients undergoing single-chamber compared with dual-chamber ICD implantation were less likely to have a lower left ventricular ejection fraction (mean [SD], 30.0% [13.1%] vs 33.1% [14.3%]; *P* < .001), and more likely to be of Black or African American race (32 088 [23.8%] vs 22 082 [16.8%]; *P* < .001), have nonischemic cardiomyopathy (51 086 [37.9%] vs 38 601 [29.5%]; *P* < .001), and be undergoing dialysis (3918 [2.9%] vs 3156 [2.4%]; *P* < .001). Patients undergoing dual-chamber compared with single-chamber ICD implantation were more likely to be older (mean [SD] age, 62.5 [13.2] vs 58.0 [14.0] years; *P* < .001), have a history of cerebrovascular disease (17 468 [13.3%] vs 15 626 [11.6%]; *P* < .001), and have a QRS duration greater than 120 ms (12 651 [9.6%] vs 6903 [5.1%]; *P* < .001).

**Figure 2.**
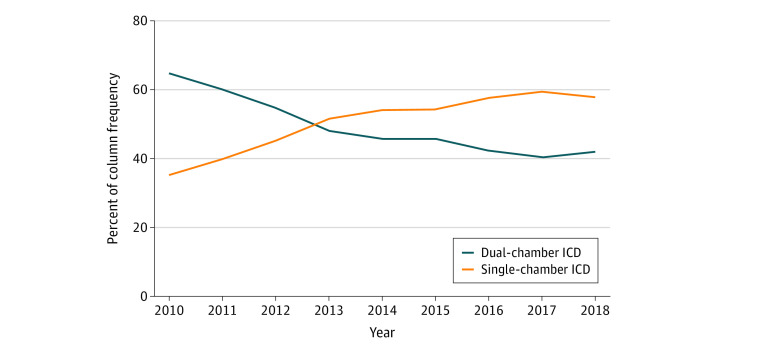
Temporal Trends in Use of Single- and Dual-Chamber Implantable Cardioverter-Defibrillators (ICDs) From 2010 to 2018

There was a significant difference between single- and dual-chamber ICD implantation based on hospital type. Private/community hospitals were more likely to implant dual-chamber devices (112 952 [51.2%] vs 107 717 [48.8%] of 220 669 patients; *P* < .001); university hospitals (24 482 [60.1%] vs 16 228 [39.9%] of 40 710 patients; *P* < .001) were more likely to implant single-chamber devices. A significant decrease in the use of dual-chamber ICDs was observed from 2010 (15 694 [64.7%]) to 2018 (9762 [42.2%]; *P* < .001) (Figure 2).

### Hospital-Level Variation

**Figure 3.**
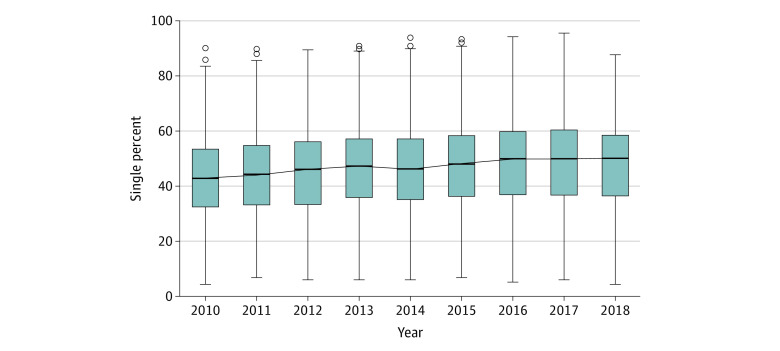
Temporally Adjusted Hospital-Level Percentage for the Use of Single-Chamber Implantable Cardioverter-Defibrillators The solid horizontal line indicates median; error bars, 95% CIs; circles, outliers.

Hospital-level variation in the use of single- and dual-chamber ICDs was present and varied throughout the study years. Following adjustments, the hospital-level median percentage of single-chamber ICD implantation increased from 42.9% (95% CI, 42.6%-45.0%) in 2010 to 50.0% (95% CI, 47.8%-51.0%) in 2018 (Figure 3). The mOR was 1.6 (95% CI, 1.6-1.8) in 2010 and 1.5 (95% CI, 1.5-1.8) in 2018 (with an mOR of 1.0 suggesting no variation between hospitals) (Table 2).

**Table 2.** Median Hospital Percentage of Single-Chamber ICD Implants and Odds Ratios Across the Study Years

Year	Median (95% CI)								
	2010	2011	2012	2013	2014	2015	2016	2017	2018
**Unadjusted**									
Odds ratio	2.6 (2.4-2.7)	2.4 (2.3-2.5)	2.3 (2.1-2.4)	2.1 (2.0-2.3)	2.1 (2.1-2.4)	2.0 (2.1-2.3)	2.4 (2.3-2.6)	2.5 (2.5-2.71)	2.5 (2.3-2.7)
Single-chamber ICD, %	37.5 (36.0-40.0)	40.0 (39.1-42.0)	46.7 (46.2-50.0)	50.0 (49.0-50.4)	55.6 (54.5-57.1)	54.2 (52.6-55.6)	58.8 (57.1-60.0)	60.0 (57.7-60.7)	58.6 (56.5-60.0)
**Adjusted**									
Odds ratio	1.6 (1.6-1.8)	1.6 (1.6-1.8)	1.6 (1.5-1.8)	1.5 (1.5-1.8)	1.6 (1.6-1.8)	1.6 (1.5-1.8)	1.6 (1.6-1.8)	1.6 (1.6-1.8)	1.5 (1.5-1.8)
Single-chamber ICD, %	42.9 (42.6-45.0)	44.4 (43.7-46.4)	46.0 (44.4-47.3)	47.3 (46.2-49.7)	46.2 (45.6-48.8)	48.2 (47.1-50.0)	50.0 (47.6-51.0)	50.0 (48.6-51.0)	50.0 (47.8-51.0)

Abbreviation: ICD, implantable cardioverter-defibrillator.

## Discussion

We evaluated the temporal trends in use and variation in single- and dual-chamber ICD implantation among patients undergoing first-time implantation without a pacing indication among a national cohort of patients treated in clinical practice. This study has several important findings. First, a significant decrease in the use of dual-chamber ICDs occurred from 2010 to 2018, but use of the dual-chamber devices remained common. Second, institutional variation in the type of device implanted was present throughout the study years, which was independent of patient characteristics. This variation suggests that institutional factors played a role in choice of device type. These results highlight the opportunity to reduce this low-value practice for patients receiving an ICD without a pacing indication.

Historically, single-chamber ICDs were used in the landmark randomized clinical trials evaluating the mortality benefit of ICDs.^1,2,3^ However, prior investigations have demonstrated substantial variation in the use of dual-chamber devices, which is independent of pacing need for patient characteristics.^4,8,9^ This variability is likely related to the complexity in the decision to implant a dual-chamber ICD for a given patient, which is dependent on a variety of clinical considerations, including the potential need for pacing in the future, effect of medications on pacing needs, arrhythmia discrimination algorithms, and specific scenarios in which pacing is beneficial (eg, long QT syndrome and some patients with hypertrophic cardiomyopathy). Furthermore, the complexity of the decision to implant an atrial lead is apparent from the appropriate use criteria whereby most clinical scenarios provide a possible appropriate rating for use of dual-chamber as opposed to single-chamber ICDs.^16^ For example, a patient with asymptomatic sinus rhythm at 55 beats per minute would otherwise not meet a pacing indication; however, when an ICD is implanted as primary prevention with an anticipated need of up-titration of β-blocker dosage, an atrial lead is a reasonable consideration for some clinicians. In addition, in at least some patients with atrial arrhythmia but not permanent atrial fibrillation, implantation of an atrial lead may be considered to aid in arrhythmia discrimination.^6^ However, we observed hospital variation in the use of single- and dual-chamber ICD implantation among this cohort that was independent of measured patient characteristics.

We also observed a difference in the association in the type of hospital and device type; private or community hospitals were more likely to implant a dual-chamber device, and university or government hospitals were more likely to implant a single-chamber device. This variation is unlikely to be explained by differences in patient characteristics based on hospital type. Despite adjusting for patient characteristics, the variation seen in this study is either owing to unmeasured patient factors or, more likely, differences in individual clinicians, local culture, clustering of graduated fellows practicing in regions where they trained, or local opinion leaders.

Differences in the care of patients that are not explained by patient needs or preferences is unwarranted variation. There are numerous examples of unwarranted variation, best described by the Dartmouth Atlas of Health Care, from both surgical and medical fields.^17^ Variation in care represents gaps in quality of care. Identifying this variation provides an opportunity for better care and renewed efforts in research and clinical quality improvement. On the basis of evidence demonstrating no improved outcomes and higher risk of complications with the addition of an atrial lead,^7,8,9^ more active approaches to changing practice may be indicated to reduce this common low-value practice among patients without a pacing indication.

The results of the present study provide a perspective on the pace and evolution of practice patterns. Although there is often a focus on adoption of newer interventions that outperform established practices, there must also be discontinuation of the use of established standards, not because a better intervention has been developed, but because what was once thought to be beneficial is not. Just as single-chamber devices were largely used in the trials evaluating the efficacy of ICDs, so was defibrillation testing at the time of implantation. Yet, the benefits of defibrillation testing have not been routinely demonstrated, several studies have shown no association between routine testing and efficacy of ICD shocks or arrhythmic death, and current guidelines provide a IIa recommendation to omit testing among selected patients.^18,19,20^ A prior analysis using the NCDR ICD Registry reported that defibrillation testing decreased substantially from 2010 to 2015, yet there was marked institutional variation.^21^ There are numerous other examples of discontinuation of the use of previously established standards, which are best seen in the evolving changes in clinical guidelines.^22,23^ Although new techniques and technology continue to advance and improve the field, further evaluation of established and routine practices would be beneficial as new insight into our understanding of patient-related outcomes becomes available.

### Limitations

This study has limitations. First, because this study was observational, we cannot exclude the possibility that unmeasured confounding variables influence the choice of device type. There are numerous clinical considerations when deciding to implant an atrial lead that are not captured in the NCDR ICD Registry. In addition, patient preference was not included in this analysis. We excluded patients undergoing subcutaneous ICD implantation, who by definition do not have a pacing indication, and this exclusion could change the ratio of dual- to single-chamber transvenous devices among centers that implant a higher volume of subcutaneous ICDs. Although the NCDR is a voluntary reporting system that encompasses the majority of ICD implantations, it is not all inclusive and it is possible that the difference in device implantation varies substantially among centers that do not report to the NCDR.

## Conclusions

In this national study of US patients undergoing first-time ICD implantation without a pacing indication, there was a significant reduction in use of dual-chamber devices over time, declining from 64.7% in 2010 to 42.2% in 2018. However, this low-value practice appears to remain common. In addition, institutional variability persists that is independent of patient characteristics in the use of atrial leads. These findings suggest differences in individual or institutional cultures of practice despite evidence and guidance that atrial leads are not necessary in the absence of a pacing indication.
